# Doxorubicin-Induced Cardiac Senescence Is Alleviated Following Treatment with Combined Polyphenols and Micronutrients through Enhancement in Mitophagy

**DOI:** 10.3390/cells12222605

**Published:** 2023-11-10

**Authors:** Eleonora Foglio, Erica D’Avorio, Laura Vitiello, Laura Masuelli, Roberto Bei, Francesca Pacifici, David Della-Morte, Simone Mirabilii, Maria Rosaria Ricciardi, Agostino Tafuri, Enrico Garaci, Matteo Antonio Russo, Marco Tafani, Federica Limana

**Affiliations:** 1 Technoscience, Parco Scientifico e Tecnologico Pontino, 04100 Latina, Italy; 2 Department of Human Sciences and Quality of Life Promotion, San Raffaele University, 00166 Rome, Italyfrancesca.pacifici@uniroma2.it (F.P.); david.dellamorte@uniroma2.it (D.D.-M.); enrico.garaci@uniroma5.it (E.G.); matteo.russo@sanraffaele.it (M.A.R.); 3 IRCCS San Raffaele Roma, 00166 Rome, Italy; laura.vitiello@sanraffaele.it; 4 Department of Experimental Medicine, Sapienza University of Rome, 00161 Rome, Italy; laura.masuelli@uniroma1.it (L.M.); marco.tafani@uniroma1.it (M.T.); 5 Department of Clinical Sciences and Translational Medicine, University of Rome “Tor Vergata”, 00133 Rome, Italy; bei@med.uniroma2.it; 6 Department of Systems Medicine, University of Rome “Tor Vergata”, 00133 Rome, Italy; 7 Department of Neurology, Evelyn F. McKnight Brain Institute, Miller School of Medicine, University of Miami, Miami, FL 33136, USA; 8 Hematology, Department of Clinical and Molecular Medicine, Sant’Andrea University Hospital, Sapienza University of Rome, 00161 Rome, Italy; simone.mirabilii@uniroma1.it (S.M.); mariarosaria.ricciardi@uniroma1.it (M.R.R.); agostino.tafuri@uniroma1.it (A.T.); 9 Laboratory of Cellular and Molecular Pathology, IRCCS San Raffaele Roma, 00166 Rome, Italy

**Keywords:** cardiac senescence, reactive oxygen species, polyphenols, mitophagy, SIRT3, SIRT4, cellular and molecular rehabilitation

## Abstract

Oxidative stress and impaired mitophagy are the hallmarks of cardiomyocyte senescence. Specifically, a decrease in mitophagic flux leads to the accumulation of damaged mitochondria and the development of senescence through increased ROS and other mediators. In this study, we describe the preventive role of A5^+^, a mix of polyphenols and other micronutrients, in doxorubicin (DOXO)-induced senescence of H9C2 cells. Specifically, H9C2 cells exposed to DOXO showed an increase in the protein expression proteins of senescence-associated genes, p21 and p16, and a decrease in the telomere binding factors TRF1 and TRF2, indicative of senescence induction. Nevertheless, A5^+^ pre-treatment attenuated the senescent-like cell phenotype, as evidenced by inhibition of all senescent markers and a decrease in SA-β-gal staining in DOXO-treated H9C2 cells. Importantly, A5^+^ restored the LC3 II/LC3 I ratio, Parkin and BNIP3 expression, therefore rescuing mitophagy, and decreased ROS production. Further, A5^+^ pre-treatment determined a ripolarization of the mitochondrial membrane and improved basal respiration. A5^+^-mediated protective effects might be related to its ability to activate mitochondrial SIRT3 in synergy with other micronutrients, but in contrast with SIRT4 activation. Accordingly, SIRT4 knockdown in H9C2 cells further increased MnSOD activity, enhanced mitophagy, and reduced ROS generation following A5^+^ pre-treatment and DOXO exposure compared to WT cells. Indeed, we demonstrated that A5^+^ protects H9C2 cells from DOXO-induced senescence, establishing a new specific role for A5^+^ in controlling mitochondrial quality control by restoring SIRT3 activity and mitophagy, which provided a molecular basis for the development of therapeutic strategies against cardiomyocyte senescence.

## 1. Introduction

The aging process is characterized by progressive structural and functional declines of the heart, and it is by far the major risk factor for the development of cardiovascular diseases (CVD) [1]. Cellular senescence is a state of irreversible cell cycle arrest associated with aging. Understanding the molecular pathways that lead to cellular senescence is critical to developing new therapeutic approaches to treat CVD [2].

Several hypotheses on the biological basis of aging have been suggested, such as oxidative stress due to increased production of reactive oxygen species (ROS) and altered mitophagy [3]. In particular, the removal of damaged mitochondria by mitophagy is essential for mitochondrial quality control and cardiac homeostasis. Disruption of this process in the heart has been linked to accelerated cardiac senescence and heart failure with the accumulation of dysfunctional mitochondria [4].

Polyphenols are naturally occurring bioactive compounds that exert important biological activities, such as antioxidant, anti-inflammatory, anti-atherogenic, and anti-aging effects, among others [5]. Different studies suggest that some natural compounds may slow down aging and protect against age-related diseases by decreasing ROS levels, limiting the progression of the senescence phenotype, and stimulating both autophagy and mitophagy [6]. The beneficial properties of the polyphenolic compound Resveratrol, including its role in the aging process and in the prevention of heart diseases, are well-known [7].

A5^+^ is a mixture composed primarily of Polydatin but also of Ellagic acid, Pterostilbene, Honokiol and other beneficial micronutrients. It has already been tested on different in vitro systems, showing a protective role against virus infections, neurodegeneration, and obesity mainly through anti-inflammatory effects [8,9,10].

In the context of senescence, Polydatin, a natural precursor of Resveratrol, has been demonstrated to counteract pro-oxidative signals induced by senescent conditions in different systems [11,12,13]. Pterostilbene reduced senescence-associated markers and postponed hepatocyte senescence [14]. Further, it attenuated oxidative stress, inflammation, and aging in keratinocytes [15]. Interestingly, Honokiol was effective in protecting cardiomyocytes against DOXO-stimulated senescence. This protective effect was mediated by inhibiting thireodoxin interactin protein (TXNIP) expression and suppressing the NLRP3 inflammasome [16].

Sirtuins are a family of histone deacetylases (HDACs) that catalyze deacetylation of both histone and non-histone lysine residues [17]. Among the seven homologs (SIRT1-7) identified to date, mammalian SIRTs have the potential to delay the onset of age-related diseases and increase mitochondrial biogenesis [18]. Moreover, their stimulation can have numerous protective effects against cardiovascular disease and could extend health and lifespan [19,20].

In this study, we hypothesized that A5^+^ could ameliorate DOXO-induced cell senescence by promoting autophagy/mitophagy and subsequently reducing oxidative stress in H9C2 cells. Further, we investigated whether the protective effect of A5^+^ might be related to sirtuin activation.

## 2. Materials and Methods

### 2.1. Cell Culture and Treatment Protocols

All the experiments in the present paper were performed on the rat embryonic ventricular cardiac cell line, H9C2 (Cat. No.88092904, Sigma-Aldrich_MERCK, St. Louis, MO, USA). H9C2 cells were maintained in 4.5 g/L glucose Dulbecco’s Modified Eagle’s Medium (DMEM) supplemented with 2 mmol/L L-glutamine, 10% heat-inactivated fetal bovine serum (FBS), and 100 units/mL penicillin, and 0.1 mg/mL streptomycin (Sigma-Aldrich_MERCK, St. Louis, MO, USA) and maintained at 37 °C in a humidified atmosphere of 5% CO_2_ and 95% air. Cells were passaged at 80% confluence with 0.25% trypsin-EDTA and used between passages 5 and 15 for all the experiments.

#### 2.1.1. Generation of SIRT4-H9C2 Silenced Cells

H9C2 cells were stably transfected with a pLKO.1 vector containing a shRNA insert to target rat SIRT4 (Cat. No. SHCLND-NM_001107147.2; Sigma-Aldrich_MERCK, St. Louis, MO, USA) (shSIRT4). Cells transfected with empty plasmids were used as controls (shControl). Briefly, 2 × 10^5^ cells were plated in 35 mm dishes for 24 h before shRNA treatment. The following day, the plasmid expressing shRNA Sirt4 (1 µg) was introduced into cells using FuGENE^®^ transfection reagent (Promega Corporation, Madison, WI, USA) according to the manufacturer’s protocol. The day after, puromycin dihydrochloride (Invitrogen, Thermo Fisher Scientific, Waltham, MA, USA), at a final concentration of 1.6 µg/mL, was added for the selection of stably silenced clones.

#### 2.1.2. A5^+^ and Doxorubicin Treatments

To induce a senescence-like phenotype, H9C2 cells were exposed to an incubation with sub-lethal concentrations of doxorubicin hydrochloride (0.1 µM DOXO) (Tocris Bioscience, Bio-Techne, Bristol, UK) for 24 h and 32 h, as previously reported by Spallarossa and colleagues [21].

A5^+^ (Sirtlife S.r.l., Rome, Italy) is composed of ellagic acid (20%), polydatin (98%), pterostilbene (20%), and honokiol (20%), mixed with recommended doses of zinc, selenium, and chromium. It is dissolved in DMSO (Sigma-Aldrich_MERCK, St. Louis, MO, USA) at 1 mg/mL, as previously reported by Pacifici et al. [9], and further diluted with complete DMEM, to reach the desired final concentration.

To investigate the protective effects of A5^+^ on DOXO-induced senescence, H9C2 wild-type (H9C2), shControl, and shSIRT4 cells were pre-treated with 50 µM A5^+^ for 48 h before supplementation with 0.1 µM DOXO in combination with A5^+^ for a further 24 h and then analyzed for each experiment. Since both A5^+^ and DOXO were dissolved in DMSO, an equivalent amount of vehicle was added to the control.

### 2.2. Western Blot Analysis

To obtain whole cell lysates, after treatments, H9C2 were pelleted at 1500 rpm (5 min at 4 °C) and lysed with Lysis buffer [50 mM Tris-HCl pH 7.4, 5 mM ethylenediaminetetraacetic acid (EDTA), 250 mM sodium chloride (NaCl), and 0.1% Triton^®^ X-100] freshly added with protease and phosphatase inhibitors (0.1 mMol/L Dithiothreitol (DTT), 50 mM sodium fluoride (NaF), 0.1 mM sodium orthovanadate (Na_3_VO_4_), 1 mM phenylmethylsulfonyl fluoride (PMSF) and 1× protease inhibitor cocktail) (All components were purchased from Sigma-Aldrich_MERCK, St. Louis, MO, USA). Protein concentration was determined by Bradford assay (Bio-Rad, Hercules, CA, USA) using the 96-well plater reader GloMax^®^-Multi Detection System (Promega Corporation, Madison, WI, USA).

Equal amounts of total cellular proteins (30 μg/lane) were boiled for 5 min at 95 °C, resolved by denaturating SDS-polyacrylamide gel electrophoresis, and transferred to a 0.45 μm nitrocellulose membrane (Bio-Rad, Hercules, CA, USA). After blocking with 5% non-fat dry milk (Bio-Rad, Hercules, CA, USA), membranes were incubated with the primary antibody overnight at 4 °C, followed by appropriated horseradish peroxidase-coupled secondary antibodies (Horseradish peroxidase-linked anti-mouse (NA931V) and anti-rabbit (NA934V) antibodies, GE Healthcare, Chicago, IL, USA) and developed by a chemiluminescence-based detection system (Lite Ablot^®^ TURBO; EuroClone, Milan, Italy). Densitometric analysis of the bands, relative to housekeeping proteins β-Actin or GAPDH, was determined by ImageJ Software v1.51 (NIH, Bethesda, MD, USA). 

The following primary antibodies were used for Western Blot analysis: mouse monoclonal anti-p21 (sc-6264; Santa Cruz Biotechnology, Inc., Dallas, TX, USA); mouse monoclonal anti-p16 (sc-1661; Santa Cruz Biotechnology Inc., Dallas, TX, USA); rabbit polyclonal anti-phospho-p53 (Ser15) (#9284; Cell Signaling Technology Inc., Danvers, MA, USA); rabbit polyclonal anti-total p53 (sc-6243; Santa Cruz Biotechnology, Inc., Dallas, TX, USA); rabbit monoclonal anti-phospho (Ser795) Rb (#9301; Cell Signaling Technology Inc., Danvers, MA, USA); mouse monoclonal anti-Rb (sc-102; Santa Cruz Biotechnology Inc., Dallas, TX, USA); mouse monoclonal anti-TRF1 (NB110-68281, Novus Biologicals, Centennial, CO, USA); mouse monoclonal anti-TRF2 (sc-271710; Santa Cruz Biotechnology Inc., Dallas, TX, USA); mouse monoclonal anti-cyclin D1 (sc-450; Santa Cruz Biotechnology Inc., Dallas, TX, USA); rabbit polyclonal anti-PARP1 (sc-7150; Santa Cruz Biotechnology Inc., Dallas, TX, USA); rabbit monoclonal anti-SIRT1 (#9475; Cell Signaling Technology Inc., Danvers, MA, USA); rabbit monoclonal anti-SIRT2 (#12650; Cell Signaling Technology Inc., Danvers, MA, USA); rabbit monoclonal anti-SIRT3 (#5490; Cell Signaling Technology Inc., Danvers, MA, USA); rabbit polyclonal anti-SIRT4 (S0948; Sigma-Aldrich_MERCK, St. Louis, MO, USA); mouse monoclonal anti-SIRT5 (sc-271635; Santa Cruz Biotechnology Inc., Dallas, TX, USA); rabbit monoclonal anti-SIRT6 (#12486; Cell Signaling Technology Inc., Danvers, MA, USA); mouse monoclonal anti-SIRT7 (sc-365344; Santa Cruz Biotechnology Inc., Dallas, TX, USA); rabbit monoclonal anti-acetyl (K122) SOD2/MnSOD (ab214675; Abcam, Cambridge, UK); rabbit polyclonal anti SOD2/MnSOD (ab13533; Abcam, Cambridge, UK); rabbit polyclonal anti LC3B (NB600-1384, Novus Biologicals, Centennial, CO, USA); mouse monoclonal anti-Parkin (sc-32282; Santa Cruz Biotechnology Inc., Dallas, TX, USA); mouse monoclonal anti-BNIP3 (sc-56167; Santa Cruz Biotechnology Inc., Dallas, TX, USA); mouse monoclonal anti β-Actin (A5316, Sigma-Aldrich–MERCK, St. Louis, MO, USA); mouse monoclonal anti-GAPDH (sc-137179; Santa Cruz Biotechnology Inc., Dallas, TX, USA).

### 2.3. Cell Viability Assay

In order to establish the concentration of A5^+^ able to promote beneficial effects without exerting toxic effects, H9C2 cells were treated with different concentrations of A5^+^ (1, 5, 10, 25, 50, 100, 200, and 500 µM) for 24 h. CellTiter 96^®^ AQueous Solution Cell Proliferation Assay (Promega Corporation, Madison, WI, USA) was used as a metabolic assay for cell viability. Briefly, cells were seeded in 96-well plates (5000 cells/well in a final volume of 100 µL). Following A5^+^ treatments, 20 µL of CellTiter 96^®^ Aqueous Solution was added to 100 µL of culture medium. After 2 h of incubation at 37 °C, absorbance at 490 nm (proportional to cell number) was measured with the 96-well plater reader GloMax^®^-Multi Detection System (Promega Corporation, Madison, WI, USA).

### 2.4. Cell Cycle Analysis

To evaluate cell death, the cell cycle was analyzed by flow cytometry in two different experimental sets. In the first setting, 6, 4, or 2 × 10^5^ H9C2 cells were seeded into 100 mm dishes and treated with increasing doses of A5^+^ (25 µM, 50 µM, 100 µM), respectively, for 24 h, 48 h, and 72 h; in the second setting, 2 × 10^5^ H9C2 cells were treated with 0.1 µM DOXO for 24 h or with 0.1 µM DOXO in combination with A5^+^ for 72 h, as described above. Subsequently, cells were harvested with trypsin-EDTA, washed twice with ice-cold PBS, centrifuged at 800× *g* for 5 min at 4 °C, and finally fixed with pre-cold 70% ethanol overnight at 4 °C. The following day, cells were washed with PBS and stained with 50 µg/mL Propidium Iodide (Sigma-Aldrich_MERCK, St. Louis, MO, USA) in the dark for 30 min at room temperature. Fluorescence was read by the LSRFortessa X-20 flow cytometer (Becton Dickinson, Milan, Italy). The sub-G1 fraction, which represents the total amount of apoptotic cells, was determined and analyzed through BD FACSDiva software (v. 8.0.2).

### 2.5. Glutamate Dehydrogenase Assay

Glutamate dehydrogenase (GDH) activity in control cells or in H9C2 cells treated with 10 µM, 25 µM, and 50 µM A5^+^ for 24 h was measured using a coupled enzyme assay, following the manufacturer’s instructions (MAK099, Sigma-Aldrich, MERCK, St. Louis, MO, USA). In this assay, glutamate is consumed by GDH, generating NADH as a reaction product, which, in turn, reacts with a probe to generate a colorimetric (450 nm) product proportional to the GDH activity present in the sample. One unit of GDH is the amount of enzyme that will generate 1.0 µmol of NADH per minute at pH 7.6 at 37 °C. Briefly, a total of 1 × 10^6^ cells were lysed in 40 μL of GDH assay buffer and kept for 10 min on ice. Afterward, lysates were centrifuged, and 10 μL of the supernatant was added to 40 μL of GDH buffer and 100 μL of a mix containing GDH assay buffer, developer, and glutamate. The whole mix was transferred into a 96-well plate and incubated at 37 °C for 3 min. Finally, absorbance was read at 450 nm using a Glomax multidetection system (Promega Corporation, Madison, WI, USA). The absorbance of the assay buffer was subtracted from each experimental sample.

### 2.6. Confocal Microscopy Monitoring of Mitochondrial Membrane Potential (Δψm)

Here, 5,5,6,6′-tetrachloro-1,1′,3,3′tetraethylbenzimi-dazoylcarbocyanine iodide (JC-1) dye was used for monitoring the mitochondrial membrane potential (Δψm) as an indicator of mitochondrial health (T3168; Thermo Fisher Scientific, Waltham, MA, USA). In mitochondria, this lipophilic cationic probe can enter and accumulate in a monomeric or aggregated form, depending on mitochondrial membrane potential (∆Ψm). In healthy cells, where ∆Ψm is high and mitochondria are energized and negatively charged, JC-1 spontaneously polymerizes to form red fluorescent J-aggregates. On the contrary, in unhealthy cells, where mitochondrial integrity is compromised and ∆Ψm assumes a lower value, JC-1 remains in a monomeric form (J-monomers), showing a green fluorescence emission. The fluorescence shift from red to green is an indicator of mitochondrial depolarization. 

For confocal microscopy monitoring of mitochondrial membrane potential, 5 × 10^4^ cells were grown on glass coverslips and treated with 25 µM A5^+^ or 50 µM A5^+^ for 72 h, with 0.1 µM DOXO for 24 h, and with 0.1 µM DOXO for 24 h in combination with A5^+^ for 72 h, as described above. Here, 72 h cultured, untreated cells were used as controls. After treatments, cell culture medium was removed and then replaced with warm medium (37 °C) containing 2 μM (final concentration) of JC-1 dye. After a 20 min incubation at 37 °C with 5% CO_2_, cells were washed in warm PBS (37 °C), and fluorescence was quickly observed by LSM 510 confocal microscopy (Zeiss, Jena, Germany).

### 2.7. Measurement of Mitochondrial Oxygen Consumption Rate (OCR)

Mitochondrial respiration was measured by plate respirometry using a Seahorse XFp Extracellular Flux Analyzer (Agilent Technologies, Santa Clara, CA, USA), as previously published [22]. Briefly, H9C2 cells were seeded in XFp-dedicated plates. After treatment with DOXO for 24 h in combination with A5^+^ for 48 h, culture medium was replaced with unbuffered DMEM medium, supplemented with 2 mM L-glutamine, 11 mM glucose, and 1.2 mM pyruvate, adjusted to pH 7.35 and then incubated for 30 min at 37 °C in a CO_2_-free incubator. Oxygen Consumption Rates (OCR), proportional to mitochondrial respiration, were measured for the basal state and following the sequential injection of oligomycin (1 μM), carbonyl cyanide-4-(trifluoromethoxy) phenylhydrazone (FCCP) (3 μM), and a mix of antimycin A (2 μM) and rotenone (Ant/Rot, 2 μM) (all reagents from Merck KGaA, Darmstadt, Germany) in each well, according to the Seahorse Mito Stress Test protocol [23]. Data were normalized by protein content through a Bradford assay, then analyzed through dedicated software (XF Wave 2.6.1, Agilent Technologies, Santa Clara, CA, USA).

### 2.8. SA-β-Gal Staining for the Assessment of Senescence

To detect senescent cells, senescence-associated β-galactosidase activity (SA-β-gal) associated with increased lysosomal content was investigated. Staining for SA-β-gal was performed with a Senescence Cell Histochemical Staining kit (CS0030; Sigma-Aldrich, MERCK, St. Louis, MO, USA), according to the manufacturer’s protocols. Briefly, 2 × 10^5^ H9C2 wt, shControl, and shSIRT4 cells were seeded in 100 mm diameter dishes and treated with 0.1 µM DOXO for 24 h or with 0.1 µM DOXO in combination with A5^+^ for 72 h, as described above. After treatments, the medium was removed and the H9C2 were fixed for 15 min with β-galactosidase fixative (4% paraformaldehyde) at room temperature and then washed three times in PBS at pH 6 at room temperature. Subsequently, the cells were incubated with freshly prepared pH 6 SA-β-gal staining solution (1 mg of 5-bromo-4-chloro-3-indolyl P3-D-galactoside (X-Gal) per ml of dimethylformamide/40 mM citric acid/sodium phosphate, 5 mM potassium ferrocyanide/5 mM potassium ferricyanide/150 mM NaCl/2 mM MgCl_2_) and incubated in a dry incubator at 37 °C without CO_2_ overnight. After staining, cells were washed twice with pH 6 PBS. Senescence was quantitated by visual inspection of blue/green stained cells using an inverted Zeiss IM35 (Zeiss, Jena, Germany) with a 100× magnification and a digital camera (Nikon Digital Sight DSL1, Nikon Corporation, Tokyo, Japan). The percentage of positive cells was calculated by counting the blue-stained cells out of the total cells in 20 randomized low-power fields for each sample.

### 2.9. Transmission Electron Microscopy

For ultrastructural analysis, 1 × 10^6^ H9C2 cells were seeded for 24 h before treatment with doxorubicin, A5^+^, or doxorubicin+A5^+^. After treatments, the medium was removed and the cells were fixed with 2.5% glutaraldehyde in PBS for 48 h, as previously described [24]. Cells were then post-fixed in osmium tetroxide, dehydrated with graded alcohol, and embedded in EPON 812 resin. Ultrathin sections were stained with uranyl acetate and lead citrate and observed with a TEM Philips Morgagni268D (FEI, Netherlands) at an accelerating voltage of 80 kV. Digital images were taken with Mega View imaging software (RADIUS 2.2) [25].

### 2.10. Reactive Oxygen Species Detection

ROS intracellular level was estimated using the cell-permanent 2′,7′-dichlorodihydrofluorescein diacetate (H2DCF-DA) fluorescent dye (Thermo Fisher Scientific, Waltham, MA, USA), according to the manufacturer’s instructions. H2DCF-DA is a non-fluorescent chemically reduced form of fluorescein, but in the presence of intracellular ROS, it is oxidized and converted, upon cleavage, to highly fluorescent dichlorofluorescein (DCF). Briefly, 2 × 10^5^ H9C2 wt, shControl, and shSIRT4 cells were seeded in 100 mm diameter dishes and treated with 0.1 µM DOXO for 24 h or with 0.1 µM DOXO in combination with A5^+^ for 72 h, as described above. After treatments, culture medium was discarded and replaced with fresh medium containing a final concentration of 25 µM H2DCF-DA dissolved in DMSO. After a 30 min incubation at 37 °C and 5% CO_2_, cells were collected and washed twice in HBSS/Ca/Mg (14025-092; Gibco, Budapest, Hungary). Subsequently, fluorescence was read by a CytoFlex flow cytometer (Beckman Coulter, Life Sciences, Brea, CA, USA), and median fluorescence intensity (MFI) has been considered for graphic analysis.

### 2.11. MnSOD Activity on Mitochondrial Fractions

Mitochondria were isolated using the Mitochondria Isolation Kit for Cultured Cells (Cat. 89874, Invitrogen, Thermofisher Scientific, Waltham, MA, USA), following the manufacturer’s instructions. Briefly, ~1 × 10^7^ cells for each treatment were harvested and pelleted at 850× *g* for 5 min. Then, Mitochondria Isolation Reagent A, supplemented with protease inhibitors, was added to the cells, followed, after a few minutes of incubation on ice, by the addition of 10 µL of Mitochondria Isolation Reagent B. Cells were then incubated on ice for 5 min, vortexing at maximum speed every minute, before adding Mitochondria Isolation Reagent C. Cells were centrifuged at 700× *g* for 10 min at 4 °C, and the supernatant was transferred to a new tube for an additional centrifugation step at 3000× *g* for 15 min at 4 °C to separate the cytosol fraction (supernatant) and the isolated mitochondria (pellet). In a final step, isolated mitochondria were washed with Mitochondria Isolation Reagent C and centrifuged at 12,000× *g* for 5 min to obtain a more purified fraction of mitochondria. Mitochondria were then lysated with RIPA buffer (50 mM Tris HCl, 150 mM NaCl, 1.0% (*v*/*v*) Triton^®^ X-100, 0.5% (*v*/*v*) Sodium Deoxycholate, 0.1% (*v*/*v*) SDS, pH 7.4) freshly added with protease and phosphatase inhibitors, and protein content was determined by a Bradford assay (Bio-Rad, Hercules, CA, USA), as described previously. 

Freshly lysated mitochondria were immediately prepared for measuring the MnSOD activity using the Superoxide Dismutase (SOD) Colorimetric Activity Kit (EIASODC, Invitrogen Thermofisher Scientific, Waltham, MA, USA), based on the manufacturer’s recommendation. Briefly, 10 μL of mitochondrial lysate or standards (serial dilutions of 4 mU/mL SOD) and 50 μL of substrate were added into a 96-well plate, and the absorption was recorded at 450 nm (background absorbance) using a Glomax multidetection system (Promega Corporation, Madison, WI, USA). Then, 25 µL of Xanthine Oxidase (a chromogenic detection reagent) were added to each well, and the plate was incubated at room temperature for 20 min before being read again at 450 nm. The total MnSOD activity (mU/mL) was calculated in relation to a SOD standard curve and normalized by the protein abundance in each sample.

### 2.12. Data Collection and Statistics

Parametric statistical analysis was performed using the Student’s *t*-test for two groups; two-way analysis of variance (ANOVA) was applied for multiple comparisons with Bonferroni post hoc analysis. Differences between groups were considered statistically significant at values of *p* < 0.05. Results are expressed as mean ± S.E.M. Data were analyzed on GraphPad version 9 for Windows (La Jolla, CA, USA).

## 3. Results

### 3.1. DOXO Treatment Induces Senescence Changes and Reduces the Expression of Mitochondrial Sirtuins in H9C2 Cells

In this study, we used an established cell model of doxorubicin-induced cell senescence [21]. Specifically, H9C2 cells were exposed to 0.1 µM doxorubicin (DOXO) for 24 h and 32 h.

**Figure 1:**
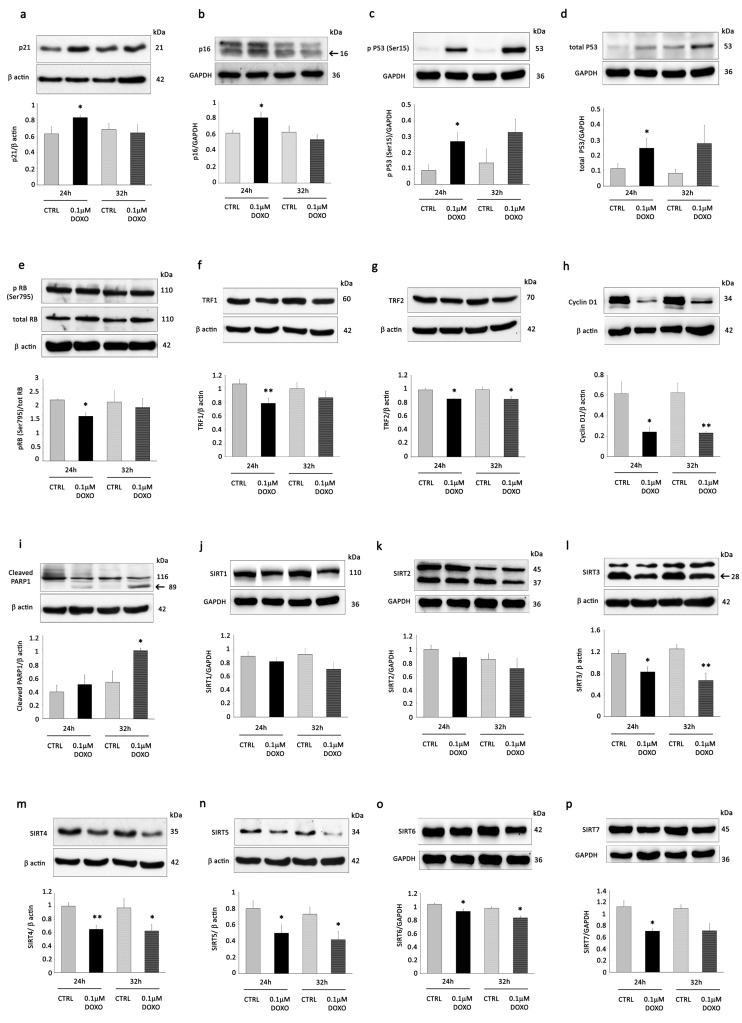
Doxorubicin (DOXO) promotes senescence and decreases the expression of sirtuins in H9C2 cells. H9C2 cells were incubated with 0.1 µM of DOXO for 24 and 32 h. Western blot analysis showing the expression of (**a**) p21, (**b**) p16, (**c**) pP53, and (**d**) total p53, (**e**) pRB/RB, (**f**) TRF1, (**g**) TRF2, (**h**) cyclin D1, (**i**) cleaved PARP1, (**j**) SIRT1, (**k**) SIRT2, (**l**) SIRT3, (**m**) SIRT4, (**n**) SIRT5, (**o**) SIRT6, (**p**) SIRT7 in H9C2 cells stimulated with 0.1 µM of DOXO for 24 and 32 h compared to control conditions (CTRL). The same filter was probed with anti-GAPDH or β-actin monoclonal antibodies to show equal loading. Upper panel: A representative Western blot of three independent experiments is shown. Lower panel: Densitometric analysis of Western blot. Data are shown as means ± SEM. * *p* < 0.05, and ** *p* < 0.01 vs. control conditions (CTRL).

To verify the induction of senescence, the protein expression of senescence-associated proteins p21 and p16 was determined by WB. Both markers were markedly increased at 24 h in the DOXO treatment group compared to the control (Figure 1a,b). In addition, at the same time point, we observed an increase in p53 phosphorylation (pP53) at Ser15, an increase in total p53 (Figure 1c,d), and a decrease in Rb phosphorylation (pRb) (Figure 1e). Interestingly, 0.1 µM DOXO significantly decreased protein levels of telomere binding factors 1 (TRF1) at 24 h (Figure 1f), while it induced a moderate (but significant) level of TRF2 downregulation at both time points (Figure 1g). These results are in agreement with previous studies showing that a moderate level of TRF2 downregulation and a consensual reduction in TRF1 expression induce senescence; otherwise, apoptosis occurs [21,26]. Accordingly, DOXO treatment determined a marked reduction in cyclin D1 protein expression (Figure 1h) but elicited Parp1 cleavage only after 32 h and not at 24 h, indicating that the induction of apoptosis in H9C2 cells was present only at the latest time point (Figure 1i).

Since sirtuins are essential factors that delay cellular senescence [27], we determined the protein expression levels of SIRT1-7. DOXO treatment caused depletion of all sirtuins (except SIRT2) mainly after 24 h (Figure 1j–p). Nevertheless, it induced a significant decrease only of mitochondrial sirtuins (i.e., SIRT3, SIRT4, SIRT5) and, to a lesser extent, of SIRT6 (Figure 1l–o) at both time points. These results are in agreement with the ability of DOXO to induce cardiac mitochondrial damage [28,29].

Based on the data obtained for senescence induction following DOXO treatment, 0.1 μM of DOXO for 24 h exposure was optimized for subsequent experiments.

### 3.2. A5^+^ Stimulates Mitochondrial SIRT3 and SIRT4, Influences Mitochondrial Activity, and Promotes Mitophagy in H9C2 Cells

Firstly, viability after A5^+^ intervention was estimated using Cell Titer and flow cytometry assays in vitro.

**Figure 2:**
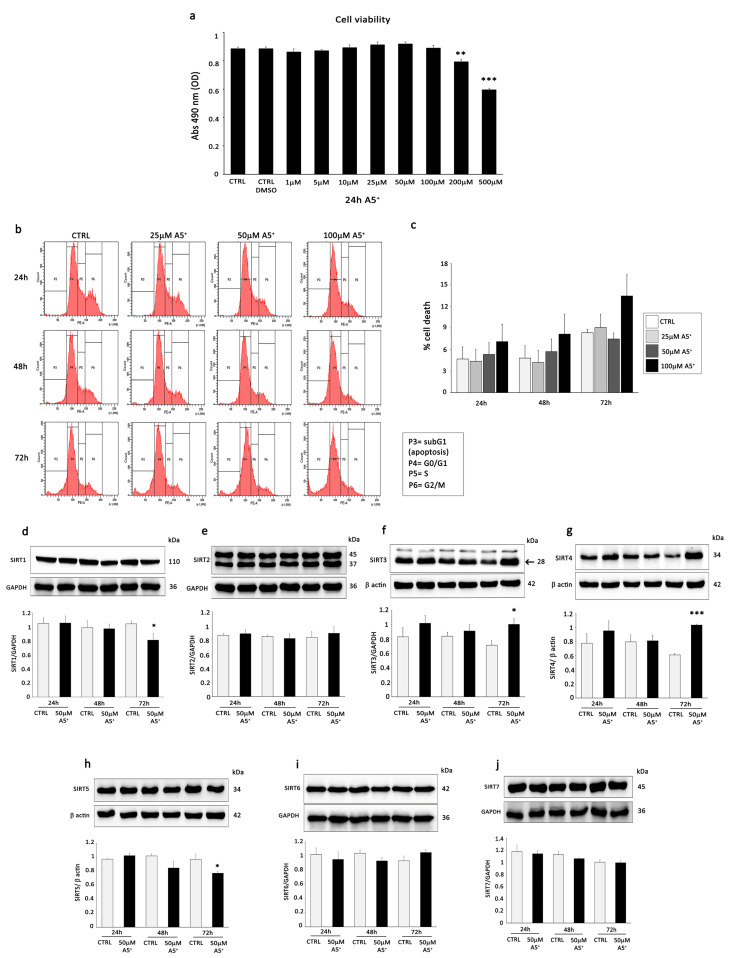
A5^+^ treatment does not impact negatively on cell viability and cell survival and modulates the expression of sirtuins in H9C2 cells. Cell viability was evaluated by the Cell Titer assay in H9C2 cells exposed for 24 h to (**a**) increasing concentration of A5^+^ (from 1 to 500 µM) vs. untreated cells. Data are expressed as the average of 490 nm Absorbance (with Absorbance proportional to cell number) from triplicate wells from 5 separate experiments. **: *p* < 0.01; ***: *p* < 0.001. Other experimental details are described in the Material and Method section. (**b**) Cell survival by the detection of apoptosis using flow cytometry. (**c**) Apoptosis analysis and the apoptotic rate were evaluated in H9C2 cells after 24, 48, and 72 h of treatment with different concentrations of A5^+^ (25, 50, and 100 µM). All results are shown as means ± SEM from three independent experiments. (**d**–**j**) Western blot analysis showing the expression of SIRT1-7 in H9C2 cells stimulated with 50 µM of A5^+^ for 24, 48, and 72 h compared to control conditions (CTRL). The same filter was probed with anti-GAPDH monoclonal Ab to show equal loading. Upper panel: A representative Western blotting of three independent experiments is shown. Lower panel: Densitometric analysis of Western blot. Data are shown as means ± SEM. * *p* < 0.05, *** *p* < 0.001 vs. control conditions (CTRL).

Importantly, exposure to a wide range of concentrations (1–500 µM) of A5^+^ for 24 h had almost no cytotoxicity to H9C2 cells. A significant decrease in cell viability was detected only at 200 and 500 µM compared to control cells (Figure 2a).

Next, we analyzed cell survival by apoptosis detection at different time points (24, 48, and 72 h) using different A5^+^ concentrations (25, 50, and 100 µM). The results of the sub-G1 assay by flow cytometry demonstrated that A5^+^ treatment did not cause a sub-G1 peak reflective of DNA fragmentation except at the latest time point, i.e., 72 h, at the highest concentration, i.e., 100 µM. (Figure 2b,c).

According to these results, we decided to use 50 µM A5^+^ for all subsequent experiments.

**Figure 3:**
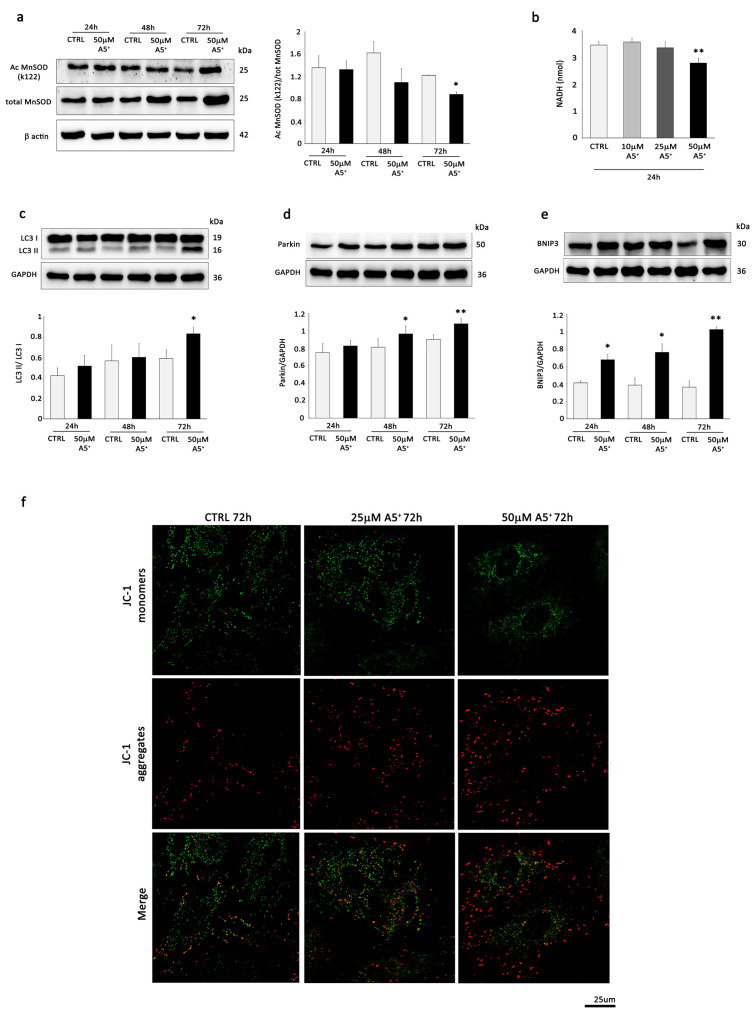
A5^+^ treatment activates the mitochondrial sirtuins SIRT3 and SIRT4 and enhances mitophagy in H9C2 cells. Western blot analysis (left panel) and relative densitometry of three independent experiments (right panel) show the expression of (**a**) K122-Ac MnSOD/MnSOD in H9C2 cells treated with 50 µM of A5^+^ for 24, 48, and 72 h compared to control conditions (CTRL). (**b**) Measurement of enzymatic activity of GDH following incubation with 10, 25, and 50 µM of A5^+^ for 24 h. The relative levels of (**c**) LC3 II/LC3 I, (**d**) Parkin, and (**e**) BNIP3 were investigated in H9C2 cells treated with 50 µM of A5^+^ for 24, 48, and 72 h by Western blot (upper panel) and relative densitometry of three independent experiments (lower panel). Data are shown as means ± SEM. * *p* < 0.05, ** *p* < 0.01, vs. control conditions (CTRL). (**f**) Assessment of mitochondrial repolarization using JC-1 dye. Increase in the red (~590 nm)/green (~529 nm) fluorescence intensity ration by exposure of H9C2 cells to A5^+^ 25 and 50 µM for 72 h.

To verify the ability of A5^+^ to modulate sirtuins, we analyzed the protein expression by WB analysis of all sirtuins following incubation of H9C2 cells with A5^+^ at 50 µM for 24, 48, and 72 h. A5^+^ treatment resulted in a significant down-modulation of SIRT1 and SIRT5 and up-regulation of mitochondrial SIRT3 and SIRT4 after 72 h of treatment (Figure 2d–j). According to these results, we sought to investigate whether SIRT3 and SIRT4 might mediate the effects of A5^+^. SIRT3 represents a major mitochondrial deacetylase, and it is well established that SIRT3 deacetylation activity protects against the development of age-related human pathology, including senescence [30,31,32]. Interestingly, A5^+^ treatment increased not only the expression of SIRT3 but also its ability to deacetylate MnSOD in H9C2 cells (Figure 3a). Specifically, as shown by WB, A5^+^ decreased the acetylation level of MnSOD compared to the control group at all time points but significantly only after 72 h of treatment.

Several studies have reported that SIRT4 inhibits the activity of glutamate dehydrogenase (GDH) enzymes, limiting the metabolism of glutamate and glutamine and, therefore, reducing ATP production [33]. We measured the activity of GDH in H9C2 cells and found that it was significantly suppressed while SIRT4 expression levels were upregulated following 50 µM A5^+^ treatment (Figure 3b).

Since mitophagy plays a vital role in regulating cardiac senescence [34], we determined the effects of A5^+^ on the cytosolic autophagosome marker LC3 B, the mitophagy kinase Parkin, and the mitophagy receptor BCL2/adenovirusE1B 19 kDa interacting protein 3 (BNIP3) in H9C2 cells compared to controls at all time points. As showed by WB analysis, stimulation with A5^+^ remarkably increased the ratio of LC3 II/LC3 I (Figure 3c) together with the protein levels of Parkin (Figure 3d) and BNIP3 (Figure 3e) in a time-dependent manner.

We next studied the changes in the mitochondria in terms of their inner membrane potential using the mitochondria-specific fluorescent probe 5,5′,6,6′-tetrachloro-1,1′,3,3′-tetraethylbenzimidazolylcarbocyanine iodide (JC-1). JC-1 is a dichromatic dye that exhibits potential-dependent accumulation in mitochondria. It exists as either a green-fluorescent monomer at depolarizing mitochondria or a red-fluorescent aggregate at polarizing mitochondria. The ratio of green to red fluorescence allows for the assessment of mitochondrial polarization states [35]. Representative photographs of JC-1-stained H9C2 cells in the absence of A5^+^ and following 72 h treatment with 25 and 50 uM A5^+^ are shown in Figure 3f. On the 3rd day, in A5^+^-treated cells, the mass of the mitochondria with a low membrane potential (green fluorescence) seemed to decrease, whereas the mass of the mitochondria with a high membrane potential (red fluorescence) seemed to increase in treated cells compared to the control.

These results suggest that A5^+^ might induce attenuation in cardiac senescence, maintaining integral mitochondrial function through mitophagy enhancement.

### 3.3. A5^+^ Alleviates Senescent-like Cell Phenotypes in Response to DOXO Treatment

To investigate whether A5^+^ mitigates DOXO-induced cell senescence, H9C2 cells were exposed to DOXO (0.1 μM) for 24 h in the absence or presence of 48 h pre-treatment with A5^+^ (50 μM).

**Figure 4:**
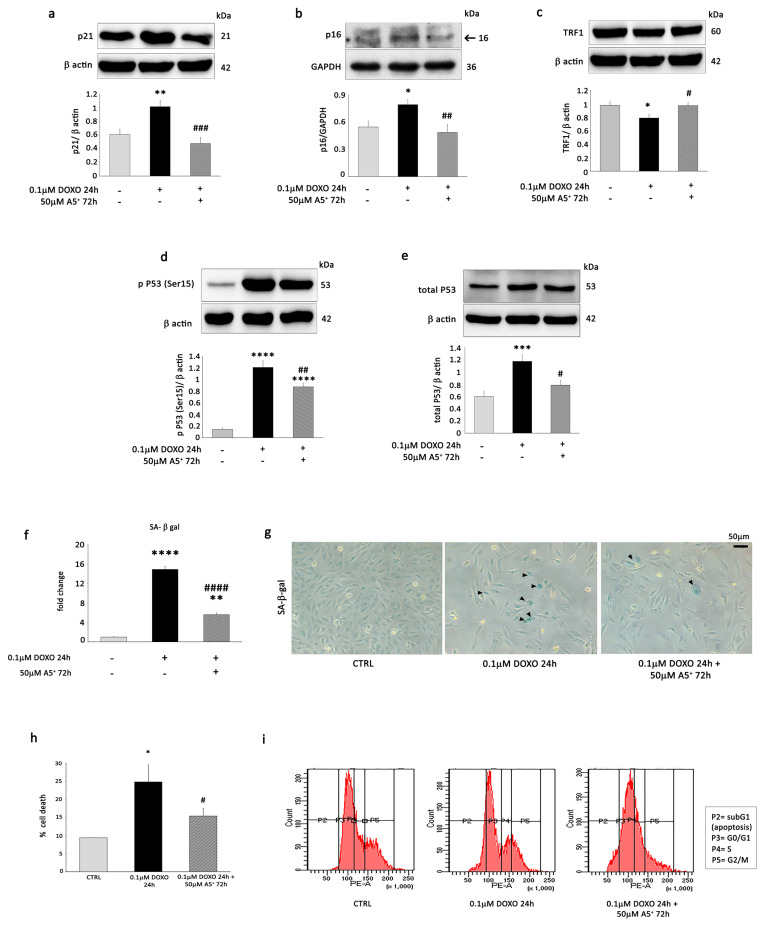
A5^+^ attenuates senescent-associated damage induced by DOXO in H9C2 cells. H9C2 cells were pre-treated with A5^+^ (50 µM) for 48 h, exposed to DOXO (0.1 µM) for the other 24 h, and subjected to WB analysis of the specific senescent markers (**a**) p21, (**b**) p16, (**c**) TRF1, (**d**) pP53, and (**e**) P53. The same filter was probed with anti-βactin and anti-GAPDH monoclonal antibodies to show equal loading. Left panel: A representative Western blot of three independent experiments is shown. Right panel: Densitometric analysis of Western blot. Data are shown as means ± SEM. * *p* < 0.05, ** *p* < 0.01, *** *p* < 0.001, **** *p* < 0.0001 vs. control conditions (CTRL), two-way ANOVA, Bonferroni post hoc test; # *p* < 0.05, ## *p* < 0.01, ### *p* < 0.001, #### *p* < 0.0001 vs. DOXO treatment, two-way ANOVA, Bonferroni post hoc test. (**f**) Percentage of SA-β-gal-positive senescent cells in cells not treated and in cells treated with DOXO (0.1 μM) in the absence or presence of A5^+^ pre-treatment (50 μM) for 48 h. (**g**) Representative images of senescence-associated-β-galactosidase (SA-β-gal) staining in H9C2 cells treated as in (**f**). Triangles indicate SA-β-gal-positive cells. (**h**) Effects of A5^+^ pre-treatment on death in DOXO-induced senescent H9C2 cells determined by cell cycle analysis. The histogram shows the proportions of cells in the sub-G1 phase. The data are from 3 independent experiments. * *p* < 0.05 vs. CTRL; # *p* < 0.05 vs. senescent DOXO-treated group. (**i**) Representative images generated by the flow cytometer.

Firstly, we analyzed the expression of the senescent markers p21 and p16. The aging markers p21 and p16 were upregulated by DOXO at the protein level, but these upregulations were inhibited by A5^+^ pre-treatment (Figure 4a,b). Previous studies identified telomere dysfunction paralleled by p53 phosphorylation and accumulation as key events in the pathogenesis of DOXO-elicited cardiomyocyte senescence [21,26]. Interestingly, we detected a reduction in TRF1 levels (Figure 4c) and an increase in those of phosphorylated and total p53 (Figure 4d,e) following incubation of H9C2 cells with doxorubicin and found that both alterations were significantly prevented by A5^+^ pre-treatment (Figure 4c,d).

Next, we tested whether A5^+^ could exert a possible effect on DOXO-induced SA-β-gal staining, a widely accepted biomarker that can distinguish senescent cells from quiescent or proliferating cells [36]. As shown in Figure 4f, pre-treating H9C2 cells with A5^+^ abrogated the enhancement in DOXO-induced H9C2 cardiomyocyte senescence. Accordingly, DOXO-induced senescent H9C2 cells displayed wide and strongly positive staining, while those that received A5^+^ pre-treatment showed less SA-β-gal staining (Figure 4g). 

To further investigate the A5^+^-mediated attenuation in cell senescence triggered by DOXO administration, cytofluorimetric analysis was also performed. Specifically, H9C2 cells were exposed for 24 h to DOXO in the absence and presence of A5^+^ in pre-treatment conditions; thereafter, PI-stained cells were analyzed by flow cytometry assay. As indicated in Figure 4h,i, senescence induction with DOXO was associated with a notable fraction of cells in the subG1 phase; nevertheless, this increment was significantly reduced in the presence of A5^+^.

These results demonstrated that DOXO-induced senescence in H9C2 cardiac cells was mitigated by A5^+^ pre-treatment.

### 3.4. A5^+^ Rescues SIRT3 and SIRT4 Expression, Reverses the Decrease in Mitophagy, and Prevents ROS Production in DOXO-Induced Senescent H9C2 Cells

**Figure 5:**
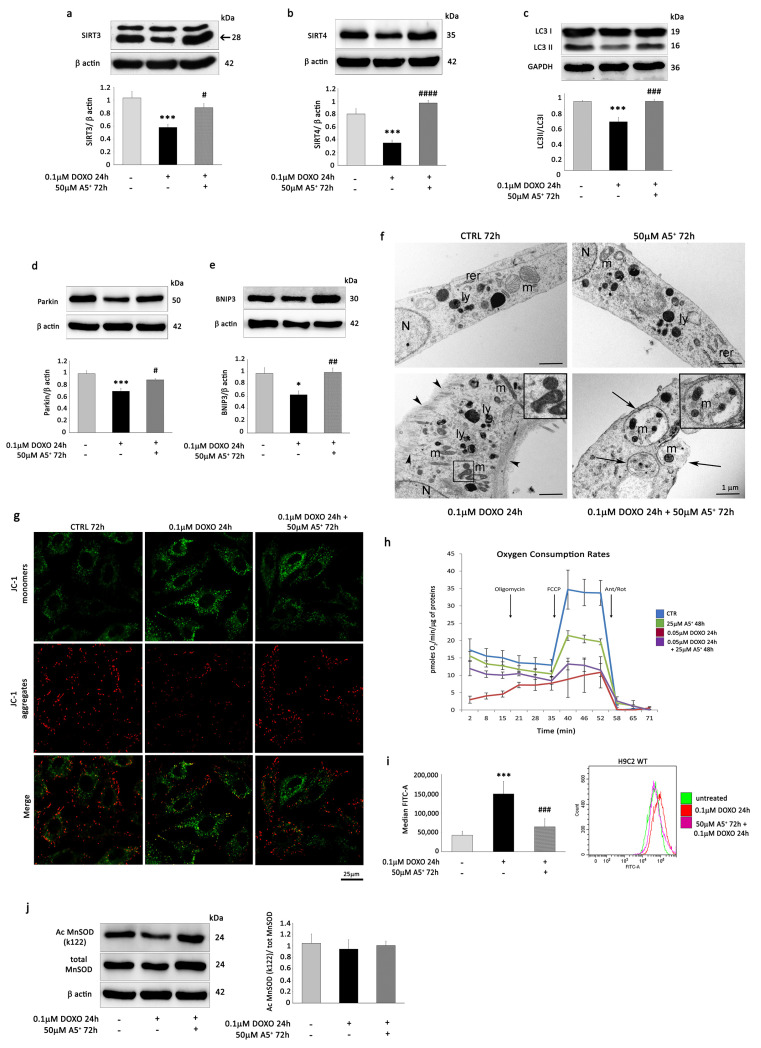
A5^+^ rescues mitophagy and attenuates ROS production in DOXO-treated H9C2 cells. After 24 h of 0.1 µM DOXO exposure with or without 48 h of 50 µM A5^+^ pre-treatment, the expression of (**a**) SIRT3, (**b**) SIRT4, (**c**) LC3 II/LC3 I, (**d**) Parkin, and (**e**) BNIP3 was determined by Western blot analysis. (**f**) Ultrastructural analysis. Upper left panel: Control H9C2 cells display an elongated appearance with normal mitochondria (m), rough endoplasmic reticulum (rer), and lysosomes (ly). Upper right panel: H9C2 cells treated with A5^+^ showed a morphology similar to that of control cells; only a slight increase in lysosomes (ly) is detectable. Bottom left panel: DOXO-treated H9C2 cells appeared bigger than control and A5^+^-treated cells. Mitochondria (m) appeared smaller. An increase in lysosomes was evident in the cytoplasm of DOXO-exposed H9C2 cells (ly). A substantial increase in the polymerization of thin and intermediate filaments below the plasma membrane and in the cytoplasm was detected (arrowheads). Bottom right panel: A5^+^-treated DOXO-exposed H9C2 cells retrieved a morphology similar to control cells. In the cytoplasm, several double membrane-surrounded vacuoles containing mitochondria, representing mitophagy, were visible (arrows; higher magnification in the square). (N: nucleus). Bars correspond to 1 µm. (**g**) The mitochondrial membrane potential was detected by JC-1 fluorescence assay (scale bar = 25 μm) in CTRL H9C2 cells (left panel), H9C2 cells exposed to 0.1 uM DOXO for 24 h (central panel) or pre-treated with 50 µM A5^+^ for 48 h and then exposed to 0.1 µM DOXO for an additional 24 h (right panel). (**h**) Time course of a Mito stress test experiment, normalized for non-mitochondrial respiration, measured through plate respirometry on CTRL H9C2 cells, H9C2 cells exposed to 0.05 uM DOXO for 24 h or pre-treated with 25 µM A5^+^ for 24 h and then exposed to 0.05 µM DOXO for an additional 24 h. Data are represented as the mean ± SD of 3 replicates. (**i**) ROS production assessed by cytofluorimetric analysis in H9C2 cells in the absence of treatment or after 24 h of 0.1 µM DOXO exposure with or without 48 h of 50 µM A5^+^ pre-treatment. *** *p* < 0.001 vs. control conditions (CTRL), ### *p* < 0.001 vs. DOXO treatment, two-way ANOVA, Bonferroni post hoc test. (**j**) WB analysis of MnSOD and acetylated K122-MnSOD in the experimental setting described above. For Western blot analysis, the same filter was probed with anti-GAPDH or β-actin monoclonal antibodies to show equal loading. Upper panel (or left panel for K122-Ac MnSOD/total MnSOD): A representative Western blotting of three independent experiments is shown. Lower panel (or right panel for Ac MnSOD/total MnSOD): Densitometric analysis of Western blot. Data are shown as means ± SEM. * *p* < 0.05, *** *p* < 0.001 vs. control conditions (CTRL), two-way ANOVA, Bonferroni post hoc test; # *p* < 0.05, ## *p* < 0.01, ### *p* < 0.001, #### *p* < 0.0001 vs. DOXO treatment, two-way ANOVA, Bonferroni post hoc test.

Since A5^+^ treatment stimulated the protein expression of mitochondrial SIRT3 and SIRT4 in H9C2 cells, we first investigated whether A5^+^ could rescue the expression of these sirtuins following DOXO exposure. WB analysis showed that 0.1 µM DOXO markedly reduced the expression of SIRT3 and SIRT4 in H9C2 cells, but 50 µM A5^+^ was able to significantly prevent the effects of DOXO on both sirtuin expressions (Figure 5a,b).

Next, we investigated whether A5^+^ abolished the DOXO-induced senescent phenotype by restoring mitophagy. DOXO treatment remarkably decreased the ratio of LC3 II/LC3 I (Figure 5c) and the mitophagy markers Parkin (Figure 5d) and BNIP3 (Figure 5e). Conversely, A5^+^ restored the LC3 II/LC3 I ratio, Parkin, and BNIP3 in H9C2-pre-treated cells compared to controls. In order to demonstrate the effect of A5^+^ pre-treatment on mitophagy in H9C2 cells following DOXO exposure, ultrastructural analysis by transmission electron microscopy was performed on H9C2 cells treated with DOXO, A5^+^, or DOXO + A5^+^ (Figure 5f).

Ultrastructural analysis of control H9C2 cells showed cells with an elongated appearance with centrally located nuclei, formed by euchromatin with poor heterochromatin and well-organized nucleoli. Mitochondria, rough endoplasmic reticulum, and few lysosomes were observed in the cytoplasm (Figure 5f, upper left panel). H9C2 cells exposed to A5^+^ were similar to control cells. No differences in subcellular organelle organization, except a slight increase in lysosomes, were detectable in the cytoplasm (Figure 5f, upper right panel). DOXO-exposed H9C2 cells appeared bigger than control and A5^+^-treated cells, although they maintained an elongated appearance with centrally located nuclei. In the cytoplasm, mitochondria appeared smaller and frequently suffered. An increase in lysosomes was evident in the cytoplasm of DOXO-exposed H9C2 cells. Moreover, a substantial increase in the polymerization of thin and intermediate filaments below the plasma membrane and in the cytoplasm was also detected (Figure 5f, bottom left panel). Notably, A5^+^-pre-treated DOXO-exposed H9C2 cells retrieved a morphology similar to that of control cells. In addition, several double membrane-surrounded vacuoles containing mitochondria, representing mitophagy, were shown in the cytoplasm, as shown in Figure 5f, bottom right panel.

Thus, ultrastructural analysis corroborated that A5^+^ promoted mitophagy in DOXO-induced senescent H9C2 cells.

To determine whether an increased clearance of damaged mitochondria by A5^+^-mediated mitophagy would improve mitochondrial function, we analyzed the effect of A5^+^ on mitochondrial membrane potential in DOXO-treated H9C2 cells using JC-1.

Interestingly, senescent cardiac cells induced by DOXO showed a depolarized mitochondrial membrane potential (Figure 5g, central panel), which was markedly reversed in the presence of A5^+^ pre-treatment, as indicated by the accumulation of JC-1 aggregates (as indicated by an increase in the amount of punctate red JC-1 fluorescence and a decrease in green monomer JC-1 fluorescence) (Figure 5g, right panel). 

Importantly, mitochondrial respiration was affected by DOXO (Figure 5h). Twenty-four hours of exposure to this agent caused a reduction in basal (−69.2 ± 6.03%, *p* = 0.006) and maximal respiration (−68.64% ± 11.17%, *p* = 0.005) when compared to vehicle (CTRL). The addition of A5^+^ improved basal respiration (A5^+^-DOXO + 58.08 ± 2.28% vs. DOXO, *p* = 0.03) but not maximal respiration (*p* = n.s.). A5^+^ alone caused a decrease of 37.08 ± 10.31% of maximal respiration when compared to vehicle (*p* = 0.04), with no effect on basal respiration (*p* = n.s.).

These results suggest that A5^+^-induced attenuation in cardiac senescence may be related to its protective effects on mitochondria and that these effects might be mediated by mitochondrial sirtuin activation.

Next, we examined ROS production in DOXO-treated H9C2 cells. As expected, DOXO increased ROS levels in H9C2, as evidenced by FACS analysis, but A5^+^ pre-treatment attenuated this increase (Figure 5i). To investigate the influence of the SIRT3 pathway on this process, we determined the acetylation status of manganese superoxide dismutase (MnSOD). Surprisingly, WB analysis did not show any decrease in K122 MnSOD acetylation following A5^+^ pre-treatment in H9C2 senescent cells (Figure 5j).

### 3.5. SIRT4 Silencing Enhances A5^+^ Protection against DOXO-Induced Senescence by Modulating Mitophagy and ROS Production

**Figure 6:**
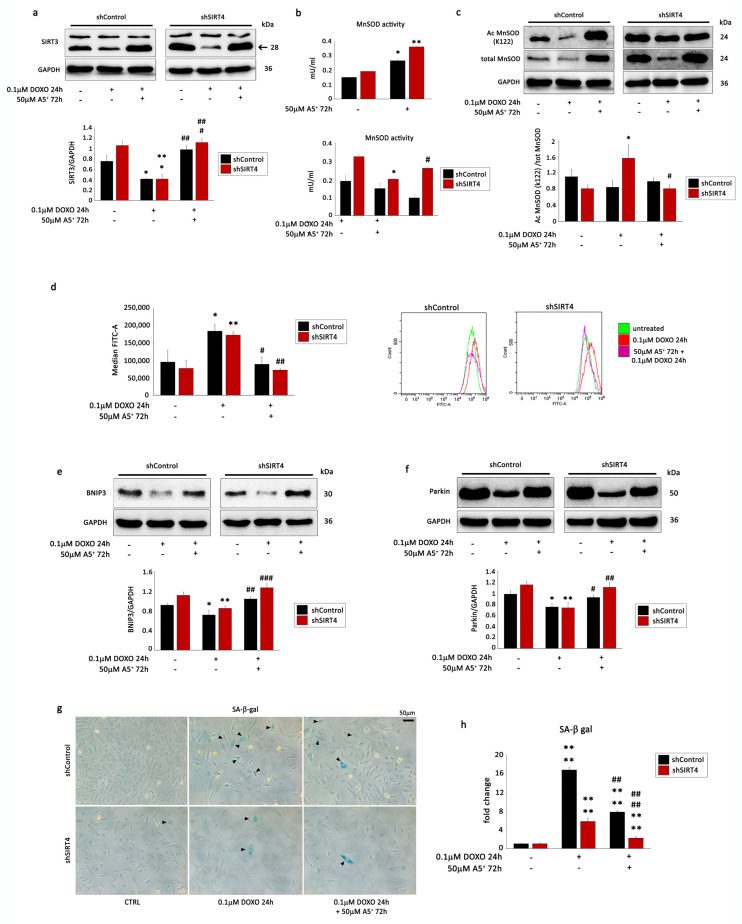
SIRT4 knockdown reverses DOXO-induced senescence in A5^+^ treated H9C2 cells by modulating mithophagy and ROS production. H9C2 cells were transfected with control shRNA (shControl) or shRNA directed against SIRT4 (shSIRT4). shControl cells and shSIRT4 cells were either left untreated or treated with DOXO (0.1 µM) for 24 h in the presence or absence of 48 h A5^+^ (50 µM) pre-treatment. WB analysis performed with antibodies against (**a**) SIRT3, (**c**) MnSOD and K122-acetylated MnSOD, (**e**) BNIP3, and (**f**) Parkin. GADPH was used as a loading control. Upper panel: A representative Western blotting of three independent experiments is shown. Lower panel: Densitometric analysis of Western blot. Data are shown as means ± SEM. * *p* < 0.05, ** *p* < 0.01 vs. untreated shControl or shSIRT4 cells, two-way ANOVA, Bonferroni post hoc test; # *p* < 0.05, ## *p* < 0.01, ### *p* < 0.001 vs. DOXO treatment in shControl or in shSIRT4 cells, two-way ANOVA, Bonferroni post hoc test. (**b**) Upper panel: MnSOD activity in shControl and shSIRT4 cells in the absence and in the presence of 50 µM A5^+^ treatment for 72 h. Lower panel: MnSOD activity in shControl and shSIRT4 cells untreated or treated with DOXO (0.1 µM) for 24 h in the presence or absence of 48 h A5^+^ (50 µM) pre-treatment. Mean ± SEM, *, *p* < 0.05, **, *p* < 0.01, vs. untreated shControl or shSIRT4 cells; #, *p* < 0.05, DOXO treatment in shSIRT4 cells. (**d**) ROS production in shControl and shSIRT4 cells was either left untreated or treated with DOXO (0.1 µM) for 24 h in the presence or absence of a 48 h A5^+^ (50 µM) pre-treatment. Median Fluorescence Intensity (MFI) was graphed on the right side. The data are representative of three separate experiments. Differences between groups were analyzed by a two-way ANOVA and a Bonferroni post hoc test. *, *p* < 0.05, **, *p* < 0.01 vs. untreated shControl or shSIRT4 cells; #, *p* < 0.05, ##, *p* < 0.01, ###, *p* < 0.001 vs. DOXO treatment in shControl or in shSIRT4 cells. (**g**) Representative images of senescence-associated-β-galactosidase (SA-β-gal) staining in shControl and shSIRT4 cells treated without or with DOXO (0.1 μM) in the absence or presence of A5^+^ pre-treatment (50 μM) for 48 h. Triangles indicate SA-β-gal-positive cells. (**h**) Percentage of SA-β-gal-positive senescent cells in cells treated as in (**g**).

Interestingly, it has been recently demonstrated that SIRT4 inhibits SIRT3-mediated MnSOD deacetylation, leading to an increase in ROS levels during cardiac hypertrophy [37]. Since A5^+^ markedly increased the protein expression of SIRT3 and SIRT4 and rescued the expression of both sirtuins following DOXO treatment, we stably silenced SIRT4 expression by shRNA. Firstly, we found that, following DOXO treatment, SIRT4 knockdown (shSIRT4) (Supplementary Figure S1a) markedly up-regulated the expression of SIRT3 (Figure 6a), while it did not have effects on the expression of the other sirtuins (Supplementary Figure S1b–f). Then, we determined SOD activity in the mitochondria of shControl and shSIRT4 H9C2 cells in the absence and in the presence of A5^+^ treatment. In basal conditions, A5^+^ and shSIRT4 cells showed higher MnSOD activity compared to shControl cells; administration of A5^+^ markedly increased this activity in both types of cells, but the increase was more significant in shSIRT4 than in shControl cells (Figure 6b, upper panel). After DOXO treatment, MnSOD activity decreased significantly in shSIRT4 cells, whereas it was still higher following A5^+^ pre-treatment and DOXO exposure. In shControl cells, all these changes were not significant (Figure 6b, lower panel).

WB analysis demonstrated that shSIRT4 reduced K122 MnSOD acetylation, confirming the inhibitory effect of SIRT4 on SIRT3-mediated deacetylation of K122 MnSOD (Figure 6c). Moreover, the reduction in ROS production in H9C2 cells pre-treated with A5^+^ following DOXO exposure was more significant in shSIRT4 H9C2 cells compared to shControl cells (Figure 6d). 

Next, we hypothesized that SIRT4 could also affect mitophagy in our experimental model. Noteworthy, the expression levels of BNIP3 and Parkin were rescued more significantly by SIRT4 knockdown following A5^+^ pre-treatment and DOXO exposure (Figure 6e,f). The LC3 II/LC3 I ratio was not rescued in H9C2-knockdown cells compared to shControl cells (Supplementary Figure S2). Accordingly, SIRT4 knockdown significantly rescued SA-β-Gal activity compared to shControl cells (Figure 6g,h). In conclusion, these results suggest that, in our in vitro model, SIRT4 silencing increased the capacity of H9C2 cells to promote mitophagy (most likely in a SIRT3-dependent manner) and therefore prevented cellular senescence, counteracting ROS generation. 

## 4. Discussion

In the present study, we demonstrated the efficacy of a mixture of polyphenols, identified as A5^+^, in preventing DOXO-induced cell senescent phenotype in vitro by promoting mitophagy and, therefore, reducing oxidative stress. These effects might be triggered, at least in part, by the A5^+^-mediated activation of SIRT3.

These findings are in agreement with previous studies demonstrating the ability of polyphenols to limit the progression of cell senescence [6]. Our mix of bioactive compounds is composed mainly of Polydatin but also of Ellagic acid, Pterostilbene, and Honokiol. Interestingly, Honokiol was effective in protecting cardiomyocytes against DOXO-stimulated senescence. This protective effect was mediated by inhibiting TXNIP expression and blocking the NLRP3 inflammasome. The resultant inhibition of inflammation resulted in the protection of cells from senescence and led to an improvement in cardiac dysfunction [16]. It is known that the presence of low-grade inflammation is a definitive characteristic of senescence progression [38], and a chronic pro-inflammatory state can aggravate cell senescence [39]. Importantly, A5^+^ has already been demonstrated to exert anti-inflammatory effects in different cell types [8,9,10]. Nevertheless, its impact on cellular senescence has never been investigated. A mix of Polydatin, Curcumin, and Quercetin was able to counteract pro-inflammatory and pro-oxidative signals induced by hyperglycemic conditions in replicative senescent HUVEC [11]. Further, a combination of Palmitoylethanolamide and Polydatin has been shown to reduce inflammation and oxidative stress in vascular injury [40]. In our in vitro system, A5^+^ attenuated DOXO-induced senescence of H9C2 cells, as demonstrated by a rescue in protein levels of the senescent markers p21 and p16. Additionally, previous findings demonstrated the crucial role of TRF1 knockdown in DOXO-stimulated cellular senescence and that this effect was mediated by p53 activity [21]. Accordingly, we found that A5^+^ pre-treatment abrogated DOXO-mediated TRF1 down-regulation and the enhancement in phosphorylated and total p53 levels.

Interestingly, metabolic analysis through plate respirometry showed that A5^+^ helped to restore some of the mitochondrial parameters that were deeply affected by the exposure to DOXO. Unexpectedly, A5^+^ alone showed an action on maximal respiration, leaving basal respiration unaltered. This peculiar mechanism of action prompts further studies on the metabolic impact of this molecule on mitochondrial metabolism.

In an attempt to identify a potential mechanism involved in A5^+^-mediated protection of H9C2 cells from DOXO-induced senescence, we focused on mitophagy since widespread targeted depletion of mitochondria through mitophagy blocks the expression of features typical of cellular senescence, such as the pro-inflammatory and pro-oxidant senescence-associated secretory phenotype and changes in the expression of the cyclin-dependent kinase inhibitors p16 and p21, while still preserving the cell cycle arrest [41]. In our study, A5^+^ treatment was able to increase the ratio of the cytosolic autophagosome kinases LC3 II/LC3 I and the expression of the mitophagic markers Parkin and BNIP3. Most importantly, A5^+^ restored mitophagy in DOXO-induced senescent H9C2 cells. The role of A5^+^ in mitophagy had important biological implications as it induced a decrease in the amount of cellular ROS, a byproduct of cell senescence [42]. In particular, BNIP3-dependent mitophagy seems to play an important role in limiting mitochondrial ROS levels [43], and, in our system, the involvement of BNIP3 in the protective effects of A5^+^ was evident in H9C2-treated cells but even more so after A5^+^ pre-treatment in DOXO-induced senescent H9C2 cells. Interestingly, it has been recently demonstrated that, in allergic rhinitis, Polydatin inhibits mitochondrial damage and mitochondrial ROS by promoting PINK1-Parkin-mediated mitophagy similarly to what we showed for A5^+^ [44].

The activation of mitophagy was paralleled by an enhancement in the expression of the mitochondrial sirtuins SIRT3 and SIRT4 and of their targets. Interestingly, it has been widely demonstrated that polyphenols like resveratrol, Pterostilbene, Polydatin and Honokiol can activate anti-inflammatory and antioxidant sirtuin pathways through the activation of autophagy and mitophagy [45,46]. In the heart, Polydatin alleviates MI and diabetic myocardial disease by maintaining mitochondrial function and enhancing autophagy by upregulating SIRT3 activity [47,48]. SIRT3 could activate mitophagy via different pathways. It promoted angiogenesis in cardiac microvascular endothelial cells by enhancing mitophagy via the PINK1/Parkin axis, thereby inhibiting ROS production and restoring vessel sprouting and tube formation [49]. Further, it could also activate mitophagy through an enhancement in BNIP3 expression, ameliorating non-alcoholic fatty liver disease [50]. Nevertheless, in the aged heart, SIRT3 promoted mitochondrial oxidative stress resistance and enhanced the ability to scavenge ROS not only by regulating mitochondrial biogenesis and mitophagy but also by deacetylating MnSOD [51,52,53]. Pre-treating H9C2 cells with A5^+^ and then exposing them to DOXO remarkably enhanced Parkin and BNIP3 expression and reduced ROS production, but it did not result in MnSOD deacetylation. It is noteworthy that in a study of angiotensin II (Ang II)-induced cardiac hypertrophy in mice, it was demonstrated that SIRT4 inhibited the binding of MnSOD to SIRT3, resulting in increased MnSOD acetylation and elevated ROS accumulation upon Ang II stimulation [37]. Taking into account these findings, we silenced SIRT4 in our cells and, accordingly, detected, in the presence of A5^+^ pre-treatment followed by DOXO exposure, a significant decrease in MnSOD acetylation compared to shControl cells, confirming the inhibitory effect of SIRT4 on SIRT3-mediated deacetylation of MnSOD. Importantly, in shSIRT4 cells, MnSOD activity was already higher in basal conditions compared to shControl cells, increased following A5^+^ treatment, and remained high after A5^+^ pre-treatment and DOXO exposure. Accordingly, the inhibition of ROS production was more significant in SIRT4-knockdown H9C2 cells compared to shControl cells.

Noteworthy, SIRT4 silencing also determined further activation of mitophagy, as evidenced by enhanced expression of Parkin and BNIP3. SIRT4 has already been reported to negatively regulate Parkin-mediated mitophagy. Specifically, moderate overexpression of SIRT4 in fibroblast models of senescence increased the levels of the inner-membrane-bound long form of the GTPase OPA1 that promoted mitochondrial dysfunction and decreased Parkin-associated mitophagy [54]. Further, in the same study, SIRT4 overexpression resulted in increased mitochondrial ROS levels and autophagic flux. Interestingly, as shown previously, in our in vitro system, SIRT4 knockdown H9C2 cells upon stimulation with A5^+^ and DOXO showed a more significant decrease in the amount of cellular ROS and no significant increase in autophagy compared to shControl cells. Together, these results suggested that SIRT4 might have an important role in managing the players involved in the antioxidant response in senescent H9C2 cells, in particular by increasing ROS production through inhibition of SIRT3-mediated MnSOD deacetylation and through a decrease in mitophagy. 

One of the limitations of the present work is that the H9C2 cell line is phenotypically distinct from cardiac myocytes, even though they share several properties [55,56]. This myoblastic cell line shows similar morphological characteristics to immature embryonic cardiomyocytes; nevertheless, it has been demonstrated that they retain several electrical and hormonal signaling pathways found in adult cardiomyocytes [57,58]. Further, it has already been used as a model to study DOXO-induced nuclear, mitochondrial, and cytoskeletal alterations [59] and DOXO-induced cell senescence [21,26].

**Figure 7:**
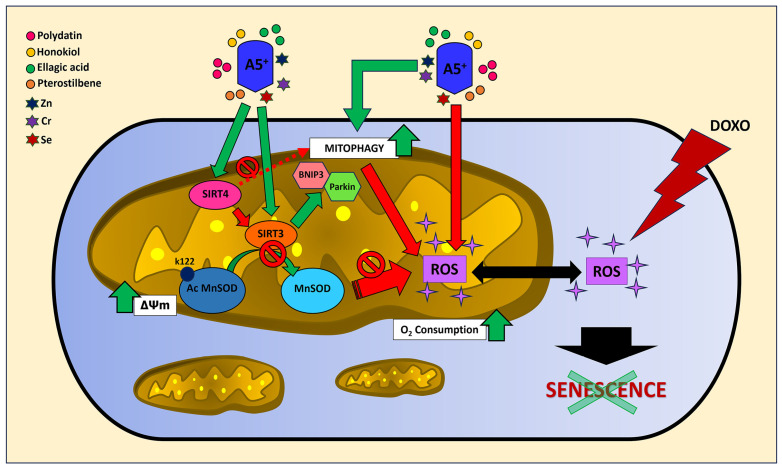
A5^+^-mediated downstream events attenuate DOXO-induced H9C2 cell senescence.

In conclusion, A5^+^ has shown the benefit of ROS scavenging and mitophagy stimulation in preventing H9C2 cell senescence but has also evidenced that simultaneous activation of mitochondrial SIRT3 and SIRT4 might not always be synergistic, as evidenced in our in vitro model (Figure 7). Nevertheless, we detected a decrease in ROS production and an increase in mitophagy following pre-treatment with A5^+^ and DOXO stimulation in shControl cells, even though to a lesser extent, and these effects, most likely, might be due to the potential synergistic effects of SIRT3 with other micronutrients present in A5^+^. For instance, zinc has already been demonstrated to induce mitophagy, leading to attenuation of mitochondrial superoxide generation in the setting of hypoxia/reoxygenation in cardiac cells [60]. Selenium, as well, has shown cardioprotective effects on AGE-induced heart failure by suppressing ROS-mediated myocyte apoptosis [61]. Nevertheless, it is important to emphasize that SIRT3 and SIRT4 opposite effects in DOXO-induced cardiomyocyte senescence might suggest one of the reasons why both short- and long-term clinical trials have failed to consistently support the cardioprotective effects of supplemental antioxidant intake in cardiomyocytes [62,63].

## Data Availability

The datasets generated during and/or analyzed during the current study are available from the corresponding author on reasonable request.

## Supplementary Materials

The following supporting information can be downloaded at: https://www.mdpi.com/article/10.3390/cells12222605/s1, Supplementary Figure S1: SIRT4 knockdown does not affect the expression of other sirtuins except SIRT3. WB analysis was performed on shControl and shSIRT4 cells with antibodies against (**a**) SIRT4, (**b**) SIRT1, (**c**) SIRT2, (**d**) SIRT5, (**e**) SIRT6, and (**f**) SIRT7 to verify the efficacy of SIRT4 knockdown and to evaluate the modulation of the expression of other sirtuins as a consequence of SIRT4 silencing. GADPH was used as a loading control. Upper panel: A representative Western blotting of three independent experiments is shown. Lower panel: Densitometric analysis of Western blot. The data are shown as means ± SEM. ***, *p* < 0.001 vs. shSIRT4 cells. Supplementary Figure S2: SIRT4 knockdown effects on LC3 II/LC3 I expression. H9C2 cells were transfected with control shRNA (shControl) or shRNA directed against SIRT4 (shSIRT4). shControl and shSIRT4 cells were either left untreated or treated with DOXO (0.1 µM) for 24 h in the presence or absence of 48 h A5^+^ (50 µM) pre-treatment. WB analysis performed with antibodies against LC3. GADPH was used as a loading control. Upper panel: A representative Western blotting of three independent experiments is shown. Lower panel: Densitometric analysis of Western blot. Data are shown as means ± SEM. * *p* < 0.05, ** *p* < 0.01, *** *p* < 0.001 vs. untreated shControl or shSIRT4 cells, two-way ANOVA, Bonferroni post hoc test; # *p* < 0.05, ## *p* < 0.01, ### *p* < 0.001 vs. DOXO treatment in shControl or in shSIRT4 cells, two-way ANOVA, Bonferroni post hoc test.

## References

[1] Chiao Y.A., Rabinovitch P.S. (2015). The Aging Heart. Cold Spring Harb. Perspect. Med..

[2] Chen M.S., Lee R.T., Garbern J.C. (2022). Senescence Mechanisms and Targets in the Heart. Cardiovasc. Res..

[3] Dai D.-F., Chen T., Johnson S.C., Szeto H., Rabinovitch P.S. (2012). Cardiac Aging: From Molecular Mechanisms to Significance in Human Health and Disease. Antioxid. Redox Signal..

[4] Kubli D.A., Quinsay M.N., Gustafsson A.B. (2013). Parkin Deficiency Results in Accumulation of Abnormal Mitochondria in Aging Myocytes. Commun. Integr. Biol..

[5] Andriantsitohaina R., Auger C., Chataigneau T., Étienne-Selloum N., Li H., Martínez M.C., Schini-Kerth V.B., Laher I. (2012). Molecular Mechanisms of the Cardiovascular Protective Effects of Polyphenols. Br. J. Nutr..

[6] Rysz J., Franczyk B., Rysz-Górzyńska M., Gluba-Brzózka A. (2021). Ageing, Age-Related Cardiovascular Risk and the Beneficial Role of Natural Components Intake. Int. J. Mol. Sci..

[7] Pyo I.S., Yun S., Yoon Y.E., Choi J.-W., Lee S.-J. (2020). Mechanisms of Aging and the Preventive Effects of Resveratrol on Age-Related Diseases. Molecules.

[8] De Angelis M., Della-Morte D., Buttinelli G., Di Martino A., Pacifici F., Checconi P., Ambrosio L., Stefanelli P., Palamara A.T., Garaci E. (2021). Protective Role of Combined Polyphenols and Micronutrients against Influenza A Virus and SARS-CoV-2 Infection In Vitro. Biomedicines.

[9] Pacifici F., Salimei C., Pastore D., Malatesta G., Ricordi C., Donadel G., Bellia A., Rovella V., Tafani M., Garaci E. (2022). The Protective Effect of a Unique Mix of Polyphenols and Micronutrients against Neurodegeneration Induced by an In Vitro Model of Parkinson’s Disease. Int. J. Mol. Sci..

[10] Pacifici F., Malatesta G., Mammi C., Pastore D., Marzolla V., Ricordi C., Chiereghin F., Infante M., Donadel G., Curcio F. (2023). A Novel Mix of Polyphenols and Micronutrients Reduces Adipogenesis and Promotes White Adipose Tissue Browning via UCP1 Expression and AMPK Activation. Cells.

[11] Matacchione G., Valli D., Silvestrini A., Giuliani A., Sabbatinelli J., Giordani C., Coppari S., Rippo M.R., Albertini M.C., Olivieri F. (2022). Curcumin, Polydatin and Quercetin Synergistic Activity Protects from High-Glucose-Induced Inflammation and Oxidative Stress. Antioxidants.

[12] Wang J., Huang C., Lin Z., Pan X., Chen J., Zheng G., Tian N., Yan Y., Zhang Z., Hu J. (2018). Polydatin Suppresses Nucleus Pulposus Cell Senescence, Promotes Matrix Homeostasis and Attenuates Intervertebral Disc Degeneration in Rats. J. Cell. Mol. Med..

[13] Wen H., Gao X., Qin J. (2014). Probing the Anti-Aging Role of Polydatin in Caenorhabditis Elegans on a Chip. Integr. Biol..

[14] Jiang Y., Zhou Y., Xu W., Wang X., Jin H., Bao X., Lu C. (2021). Induction of Sestrin2 by Pterostilbene Suppresses Ethanol-Triggered Hepatocyte Senescence by Degrading CCN1 via P62-Dependent Selective Autophagy. Cell Biol. Toxicol..

[15] Teng W.-L., Huang P.-H., Wang H.-C., Tseng C.-H., Yen F.-L. (2021). Pterostilbene Attenuates Particulate Matter-Induced Oxidative Stress, Inflammation and Aging in Keratinocytes. Antioxidants.

[16] Huang P.-P., Fu J., Liu L.-H., Wu K.-F., Liu H.-X., Qi B.-M., Liu Y., Qi B.-L. (2020). Honokiol Antagonizes Doxorubicin-induced Cardiomyocyte Senescence by Inhibiting TXNIP-mediated NLRP3 Inflammasome Activation. Int. J. Mol. Med..

[17] Seto E., Yoshida M. (2014). Erasers of Histone Acetylation: The Histone Deacetylase Enzymes. Cold Spring Harb. Perspect. Biol..

[18] van de Ven R.A.H., Santos D., Haigis M.C. (2017). Mitochondrial Sirtuins and Molecular Mechanisms of Aging. Trends Mol. Med..

[19] Matsushima S., Sadoshima J. (2015). The Role of Sirtuins in Cardiac Disease. Am. J. Physiol. Heart Circ. Physiol..

[20] Kane A.E., Sinclair D.A. (2018). Sirtuins and NAD+ in the Development and Treatment of Metabolic and Cardiovascular Diseases. Circ. Res..

[21] Spallarossa P., Altieri P., Aloi C., Garibaldi S., Barisione C., Ghigliotti G., Fugazza G., Barsotti A., Brunelli C. (2009). Doxorubicin Induces Senescence or Apoptosis in Rat Neonatal Cardiomyocytes by Regulating the Expression Levels of the Telomere Binding Factors 1 and 2. Am. J. Physiol. Heart Circ. Physiol..

[22] Allegretti M., Ricciardi M.R., Licchetta R., Mirabilii S., Orecchioni S., Reggiani F., Talarico G., Foà R., Bertolini F., Amadori S. (2015). The Pan-Class I Phosphatidyl-Inositol-3 Kinase Inhibitor NVP-BKM120 Demonstrates Anti-Leukemic Activity in Acute Myeloid Leukemia. Sci. Rep..

[23] Divakaruni A.S., Paradyse A., Ferrick D.A., Murphy A.N., Jastroch M. (2014). Analysis and Interpretation of Microplate-Based Oxygen Consumption and PH Data. Methods in Enzymology.

[24] Modesti A., Masuelli L., Modica A., D’Orazi G., Scarpa S., Bosco M.C., Forni G. (1993). Ultrastructural Evidence of the Mechanisms Responsible for Interleukin-4-Activated Rejection of a Spontaneous Murine Adenocarcinoma. Int. J. Cancer.

[25] Franchini V., De Sanctis S., Marinaccio J., De Amicis A., Coluzzi E., Di Cristofaro S., Lista F., Regalbuto E., Doria A., Giovenale E. (2018). Study of the Effects of 0.15 Terahertz Radiation on Genome Integrity of Adult Fibroblasts. Environ. Mol. Mutagen..

[26] Altieri P., Barisione C., Lazzarini E., Garuti A., Bezante G.P., Canepa M., Spallarossa P., Tocchetti C.G., Bollini S., Brunelli C. (2016). Testosterone Antagonizes Doxorubicin-Induced Senescence of Cardiomyocytes. J. Am. Heart Assoc..

[27] Lee S.-H., Lee J.-H., Lee H.-Y., Min K.-J. (2019). Sirtuin Signaling in Cellular Senescence and Aging. BMB Rep..

[28] He L., Liu F., Li J. (2021). Mitochondrial Sirtuins and Doxorubicin-Induced Cardiotoxicity. Cardiovasc. Toxicol..

[29] Wallace K.B., Sardão V.A., Oliveira P.J. (2020). Mitochondrial Determinants of Doxorubicin-Induced Cardiomyopathy. Circ. Res..

[30] Wang X.-Q., Shao Y., Ma C.-Y., Chen W., Sun L., Liu W., Zhang D.-Y., Fu B.-C., Liu K.-Y., Jia Z.-B. (2014). Decreased SIRT3 in Aged Human Mesenchymal Stromal/Stem Cells Increases Cellular Susceptibility to Oxidative Stress. J. Cell. Mol. Med..

[31] Ma C., Sun Y., Pi C., Wang H., Sun H., Yu X., Shi Y., He X. (2020). Sirt3 Attenuates Oxidative Stress Damage and Rescues Cellular Senescence in Rat Bone Marrow Mesenchymal Stem Cells by Targeting Superoxide Dismutase 2. Front. Cell Dev. Biol..

[32] Li P., Newhardt M.F., Matsuzaki S., Eyster C., Pranay A., Peelor F.F., Batushansky A., Kinter C., Subramani K., Subrahmanian S. (2023). The Loss of Cardiac SIRT3 Decreases Metabolic Flexibility and Proteostasis in an Age-Dependent Manner. GeroScience.

[33] Haigis M.C., Mostoslavsky R., Haigis K.M., Fahie K., Christodoulou D.C., Murphy A.J., Valenzuela D.M., Yancopoulos G.D., Karow M., Blander G. (2006). SIRT4 Inhibits Glutamate Dehydrogenase and Opposes the Effects of Calorie Restriction in Pancreatic Beta Cells. Cell.

[34] Liang W.J., Gustafsson Å.B. (2020). The Aging Heart: Mitophagy at the Center of Rejuvenation. Front. Cardiovasc. Med..

[35] Sivandzade F., Bhalerao A., Cucullo L. (2019). Analysis of the Mitochondrial Membrane Potential Using the Cationic JC-1 Dye as a Sensitive Fluorescent Probe. Bio-Protocol.

[36] Dimri G.P., Lee X., Basile G., Acosta M., Scott G., Roskelley C., Medrano E.E., Linskens M., Rubelj I., Pereira-Smith O. (1995). A Biomarker That Identifies Senescent Human Cells in Culture and in Aging Skin in Vivo. Proc. Natl. Acad. Sci. USA.

[37] Luo Y.-X., Tang X., An X.-Z., Xie X.-M., Chen X.-F., Zhao X., Hao D.-L., Chen H.-Z., Liu D.-P. (2017). SIRT4 Accelerates Ang II-Induced Pathological Cardiac Hypertrophy by Inhibiting Manganese Superoxide Dismutase Activity. Eur. Heart J..

[38] Minguzzi M., Cetrullo S., D’Adamo S., Silvestri Y., Flamigni F., Borzì R.M. (2018). Emerging Players at the Intersection of Chondrocyte Loss of Maturational Arrest, Oxidative Stress, Senescence and Low-Grade Inflammation in Osteoarthritis. Oxid. Med. Cell. Longev..

[39] Del Pinto R., Ferri C. (2018). Inflammation-Accelerated Senescence and the Cardiovascular System: Mechanisms and Perspectives. Int. J. Mol. Sci..

[40] Gugliandolo E., Fusco R., Biundo F., D’Amico R., Benedetto F., Di Paola R., Cuzzocrea S. (2017). Palmitoylethanolamide and Polydatin Combination Reduces Inflammation and Oxidative Stress in Vascular Injury. Pharmacol. Res..

[41] Herranz N., Gil J. (2016). Mitochondria and Senescence: New Actors for an Old Play. EMBO J..

[42] De Gaetano A., Gibellini L., Zanini G., Nasi M., Cossarizza A., Pinti M. (2021). Mitophagy and Oxidative Stress: The Role of Aging. Antioxidants.

[43] Li J., Lin Q., Shao X., Li S., Zhu X., Wu J., Mou S., Gu L., Wang Q., Zhang M. (2023). HIF1α-BNIP3-Mediated Mitophagy Protects against Renal Fibrosis by Decreasing ROS and Inhibiting Activation of the NLRP3 Inflammasome. Cell Death Dis..

[44] Liu S., Wang C., Zhang Y., Zhang Y., Song Y., Jiang J., Liu R., Jin H., Yan G., Jin Y. (2023). Polydatin Inhibits Mitochondrial Damage and Mitochondrial ROS by Promoting PINK1-Parkin-Mediated Mitophagy in Allergic Rhinitis. FASEB J..

[45] Wan W., Hua F., Fang P., Li C., Deng F., Chen S., Ying J., Wang X. (2022). Regulation of Mitophagy by Sirtuin Family Proteins: A Vital Role in Aging and Age-Related Diseases. Front. Aging Neurosci..

[46] Zheng Y., Shi B., Ma M., Wu X., Lin X. (2019). The Novel Relationship between Sirt3 and Autophagy in Myocardial Ischemia–Reperfusion. J. Cell. Physiol..

[47] Zhang M., Zhao Z., Shen M., Zhang Y., Duan J., Guo Y., Zhang D., Hu J., Lin J., Man W. (2017). Polydatin Protects Cardiomyocytes against Myocardial Infarction Injury by Activating Sirt3. Biochim. Biophys. Acta Mol. Basis Dis..

[48] Zhang M., Wang S., Cheng Z., Xiong Z., Lv J., Yang Z., Li T., Jiang S., Gu J., Sun D. (2017). Polydatin Ameliorates Diabetic Cardiomyopathy via Sirt3 Activation. Biochem. Biophys. Res. Commun..

[49] Wei T., Huang G., Gao J., Huang C., Sun M., Wu J., Bu J., Shen W. (2017). Sirtuin 3 Deficiency Accelerates Hypertensive Cardiac Remodeling by Impairing Angiogenesis. J. Am. Heart Assoc..

[50] Li R., Xin T., Li D., Wang C., Zhu H., Zhou H. (2018). Therapeutic Effect of Sirtuin 3 on Ameliorating Nonalcoholic Fatty Liver Disease: The Role of the ERK-CREB Pathway and Bnip3-Mediated Mitophagy. Redox Biol..

[51] Zhao D., Sun Y., Tan Y., Zhang Z., Hou Z., Gao C., Feng P., Zhang X., Yi W., Gao F. (2018). Short-Duration Swimming Exercise after Myocardial Infarction Attenuates Cardiac Dysfunction and Regulates Mitochondrial Quality Control in Aged Mice. Oxid. Med. Cell. Longev..

[52] Parodi-Rullán R.M., Chapa-Dubocq X.R., Javadov S. (2018). Acetylation of Mitochondrial Proteins in the Heart: The Role of SIRT3. Front. Physiol..

[53] Tomczyk M.M., Cheung K.G., Xiang B., Tamanna N., Fonseca Teixeira A.L., Agarwal P., Kereliuk S.M., Spicer V., Lin L., Treberg J. (2022). Mitochondrial Sirtuin-3 (SIRT3) Prevents Doxorubicin-Induced Dilated Cardiomyopathy by Modulating Protein Acetylation and Oxidative Stress. Circ. Heart Fail..

[54] Lang A., Anand R., Altinoluk-Hambüchen S., Ezzahoini H., Stefanski A., Iram A., Bergmann L., Urbach J., Böhler P., Hänsel J. (2017). SIRT4 Interacts with OPA1 and Regulates Mitochondrial Quality Control and Mitophagy. Aging.

[55] Hescheler J., Meyer R., Plant S., Krautwurst D., Rosenthal W., Schultz G. (1991). Morphological, Biochemical, and Electrophysiological Characterization of a Clonal Cell (H9c2) Line from Rat Heart. Circ. Res..

[56] Kimes B.W., Brandt B.L. (1976). Properties of a Clonal Muscle Cell Line from Rat Heart. Exp. Cell Res..

[57] Brostrom M.A., Reilly B.A., Wilson F.J., Brostrom C.O. (2000). Vasopressin-Induced Hypertrophy in H9c2 Heart-Derived Myocytes. Int. J. Biochem. Cell Biol..

[58] Wayman N., McDonald M.C., Thompson A.S., Threadgill M.D., Thiemermann C. (2001). 5-Aminoisoquinolinone, a Potent Inhibitor of Poly (Adenosine 5’-Diphosphate Ribose) Polymerase, Reduces Myocardial Infarct Size. Eur. J. Pharmacol..

[59] Sardão V.A., Oliveira P.J., Holy J., Oliveira C.R., Wallace K.B. (2009). Morphological Alterations Induced by Doxorubicin on H9c2 Myoblasts: Nuclear, Mitochondrial, and Cytoskeletal Targets. Cell Biol. Toxicol..

[60] Bian X., Teng T., Zhao H., Qin J., Qiao Z., Sun Y., Liun Z., Xu Z. (2018). Zinc Prevents Mitochondrial Superoxide Generation by Inducing Mitophagy in the Setting of Hypoxia/Reoxygenation in Cardiac Cells. Free Radic. Res..

[61] Zhu H., Wang X., Meng X., Kong Y., Li Y., Yang C., Guo Y., Wang X., Yang H., Liu Z. (2022). Selenium Supplementation Improved Cardiac Functions by Suppressing DNMT2-Mediated GPX1 Promoter DNA Methylation in AGE-Induced Heart Failure. Oxid. Med. Cell. Longev..

[62] Finkel T., Holbrook N.J. (2000). Oxidants, Oxidative Stress and the Biology of Ageing. Nature.

[63] Pellegrino D. (2016). Antioxidants and Cardiovascular Risk Factors. Diseases.

