# Supplementary material for: Doxorubicin-Induced Cardiac Senescence Is Alleviated Following Treatment with Combined Polyphenols and Micronutrients through Enhancement in Mitophagy
Source: Cells. 2023 Nov 10;12(22):2605. doi: 10.3390/cells12222605 (PMC10670650; doi:10.3390/cells12222605)
Supplement: Supplementary file 1 [file cells-12-02605-s001.zip › cells-2503271-supplementary.pdf]

Supplementary Materials:

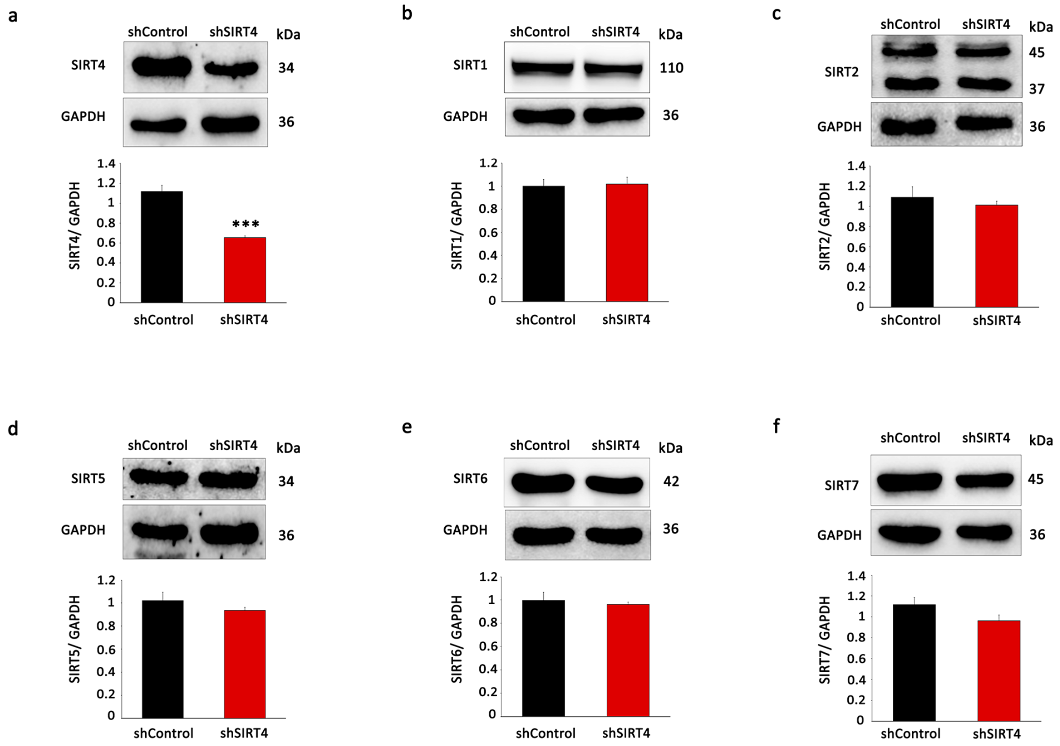

Supplementary Figure S1. SIRT4 knockdown does not affect the expression of other sirtuins except SIRT3. WB analysis was performed on shControl and shSIRT4 cells with antibodies against (a) SIRT4, (b) SIRT1, (c) SIRT2, (d) SIRT5, (e) SIRT6 and (f) SIRT7 to verify the efficacy of SIRT4 knockdown and to evaluate the modulation of the expression of other sirtuins, as a consequence of SIRT4 silencing. GADPH was used as loading control. Upper panel: A representative Western blotting of three independent experiments is shown. Lower panel: Densitometric analysis of Western blot. Data are shown as means ± SEM. ***, $P < 0.001$ vs shSIRT4 cells.

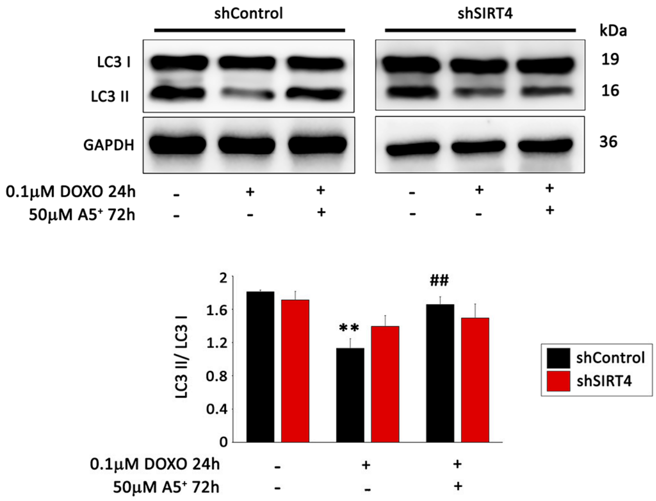

Supplementary Figure S2. SIRT4 knockdown effects on LC3 II/LC3 I expression H9C2 cells were transfected with control shRNA (shControl) or shRNA directed against SIRT4 (shSIRT4). shControl and shSIRT4 cells were either left untreated or treated with DOXO (0.1 µM) for 24h in the presence or absence of 48h A5+ (50 µM) pre-treatment. WB analysis performed with antibodies against LC3. GADPH was used as loading control. Upper panel: A representative Western blotting of three independent experiments is shown. Lower panel: Densitometric analysis of Western blot. Data are shown as means ± SEM. *, $P < 0.05$, **, $P < 0.01$, ***, $P < 0.001$ vs untreated shControl or shSIRT4 cells, two-way ANOVA, Bonferroni post hoc test; #, $P < 0.05$, ##, $P < 0.01$, ###, $P < 0.001$ vs DOXO treatment in shControl or in shSIRT4 cells, two-way ANOVA, Bonferroni post hoc test.
